# Information Scale Correction for Varying Length Amplicons Improves Eukaryotic Microbiome Data Integration

**DOI:** 10.3390/microorganisms11040949

**Published:** 2023-04-06

**Authors:** Tong Zhou, Feng Zhao, Kuidong Xu

**Affiliations:** 1 Laboratory of Marine Organism Taxonomy and Phylogeny, Qingdao Key Laboratory of Marine Biodiversity and Conservation, Institute of Oceanology, Chinese Academy of Sciences, Qingdao 266071, China; 2 Shandong Province Key Laboratory of Experimental Marine Biology, Institute of Oceanology, Chinese Academy of Sciences, Qingdao 266071, China; 3 University of Chinese Academy of Sciences, Beijing 100049, China

**Keywords:** integration, microbiome, molecular ecology, big data, information scale

## Abstract

The integration and reanalysis of big data provide valuable insights into microbiome studies. However, the significant difference in information scale between amplicon data poses a key challenge in data analysis. Therefore, reducing batch effects is crucial to enhance data integration for large-scale molecular ecology data. To achieve this, the information scale correction (ISC) step, involving cutting different length amplicons into the same sub-region, is essential. In this study, we used the Hidden Markov model (HMM) method to extract 11 different 18S rRNA gene v4 region amplicon datasets with 578 samples in total. The length of the amplicons ranged from 344 bp to 720 bp, depending on the primer position. By comparing the information scale correction of amplicons with varying lengths, we explored the extent to which the comparability between samples decreases with increasing amplicon length. Our method was shown to be more sensitive than V-Xtractor, the most popular tool for performing ISC. We found that near-scale amplicons exhibited no significant change after ISC, while larger-scale amplicons exhibited significant changes. After the ISC treatment, the similarity among the data sets improved, especially for long amplicons. Therefore, we recommend adding ISC processing when integrating big data, which is crucial for unlocking the full potential of microbial community studies and advancing our knowledge of microbial ecology.

## 1. Introduction

Big data integration and reanalysis always collect a vast number of research data sets generated by varying technology. Big data integration could be applied to bring together new and existing data to develop high-resolution models of the global environment and obtain new insights for both research and policy decision-making [1]. A recent study gathered published datasets targeting the short sub region of the eukaryotic 18S rRNA gene from abyssal and bathyal sediments and euphotic and aphotic ocean pelagic layers to reveal the patterns of eukaryotic diversity from the surface water to the deep-ocean sediment [2]. The big data integration and reanalysis provide the basis for a more informed and effective strategy for understanding the microbiome in the Earth system. 

The information scale difference between amplicon data is a critical problem. Particularly, long-read sequencing, which is selected as Technology of the Year 2022, drives genomics research. Traditional next-generation sequencing methods can only cover 1–2 hypervariable regions [3]. Full-length fragments contain more information than short ones, which allows more accurate taxonomic annotation [4,5]. However, it is challenging to compare amplicon data by different information scales. This is because the higher resolution of the long amplicon makes the abundance table longer and the shared fewer species with the short amplicon. Based on the high coverage of complete bacteria genomes, the data integration method is to map short fragments to the phylogeny tree of complete genomes, and based on the phylogeny relationship to count the abundance among samples [6]. However, there is a high rate of uncultured and incomplete genomes for the eukaryote microbiome. Therefore, it is challenging to integrate eukaryote amplicon (such as 18S rRNA gene) data by phylogeny method.

The recent study used the digital simulation methods to extract hypervariable regions from full-length sequences that are consistent with the amplicon regions of the short sequencing [7]. We called the step of cutting the different length amplicon into the same sub-region information scale correction (ISC). There are two main methods to correct the information scale: (1) in-silico PCR by primer sequences which cannot avoid the problem of amplifying the bias [8,9], and (2) the Hidden Markov model (HMM) which can avoid the dependence on the primer region. HMM-based tools are thus commonly used for sequence extraction. MitoGeneExtractor [10] and MitoFinder [11] are widely used for extracting mitochondrial COI, ITSx is used for extracting fungal ITS [12], Metaxa2 is used for extracting small and large subunit rRNA from bacteria, archaea, and fungi [13], and V-Xtractor is used for extracting various highly variable regions of the small subunit rRNA (16S/18S) sequence from bacteria, archaea, and fungi [14]. Among them, V-Xtractor can recognize and extract various hypervariable regions of the 18S rRNA gene. However, V-Xtractor’s HMM model training is based on the sequence features of the primer binding regions, rather than the complete sequence features of the hypervariable regions. For some species, there may be significant differences in the primer binding region features, which can lead to a decrease in recognition sensitivity. These types of tools must continually include more species information to supplement their model libraries [12].

To address the lack of a short primer binding region, we trained an HMM model based on the complete sequence features of the 18S rRNA gene v4 region, which can extract sequences based on complete sequence features. We used the HMM method to extract the amplicon by 11 different 18S rRNA gene v4 region primer sets. The amplicon length ranges from 344 bp to 720 bp basing the primer position. A reliable batch effect correction method should aim to avoid over-correction and correct non-sample ecological differences caused by known technical issues [15]. In the absence of other factors, if there is no significant difference in the information scale, there should be no significant change in community structure before and after correction. Comparison of information scale correction for amplicons of varying lengths explores whether the comparability between samples decreases as the length increases. Therefore, it is inferred that with the accumulation of long-read sequencing amplicon data [16], we should consider adding information scale correction as part of the big data integration analysis workflow.

## 2. Materials and Methods

### 2.1. Data Collection

We chose the 18S rRNA gene v4 region as a representative because it is the region with the most extensive usage [3,17], as well as the varying amplicon length [18]. Based on the primer information in the PR2-primer database v1.1.1 (accessed on 18 February 2023 https://app.pr2-primers.org/pr2-primers/) [18], we used the key word ‘metabarcoding’ to filter primers for the 18S rRNA gene v4 region used for metabarcoding, with a total of 85 primer pairs. Based on the literature corresponding to the primers, 11 datasets were successfully downloaded with a total of 578 samples of raw data (Table 1). Using the most used primer pairs as a baseline [3], the amplification regions are classified into four categories of ‘Same’ with 143 samples of 2 datasets, ‘Near’ with 62 samples of 3 datasets, ‘Short’ with 100 samples of 2 datasets and ‘Long’ with 292 samples of 4 datasets according to region difference. The category of the Same includes the primers which amplify the same position. The category of Near contains the primers which amplify the position with less than 50 bp difference. The Short category represents the primers those amplifying position >50 bp shorter than the baseline region, and the Long category means the primers those amplifying position >50 bp longer than the baseline region. Regarding data selection, we tried to ensure that each amplicon category had at least two datasets, and each dataset had a sample size of at least 10, in order to have a sufficient number of samples for comparing the differences in community structure before and after ISC treatment.

We utilized a naming convention that assigned unique group ids to different groups, and sample ids were used to rename the obtained samples, not ENA/SRA ids. To process the data, we used the fastq-dump function of SRA-tools software v 3.0.3 (accessed on 18 February 2023 https://github.com/ncbi/sra-tools) to split the SRA files for Illumina MiSeq paired-end sequencing data, while 454 sequencing data were directly converted to fastq files. The data were then merged and resampled using USEARCH v 11.0.667_i86linux64 (accessed on 1 September 2021 https://drive5.com/usearch/) [30]. To maintain a consistent sequencing depth and minimize computational complexity, we removed samples with less than 5000 reads, and resampled samples with more than 10,000 reads to 10,000 reads by fastx_subsample function in USEARCH. This step was taken to ensure that the sequencing depth was uniform across all samples, which is crucial for reliable downstream analyses. By using these approaches, we were able to process and prepare the sequencing data for downstream analyses effectively. This enabled us to compare and evaluate the performance of different methods accurately, leading to robust and reliable findings. 

### 2.2. Information Scale Correction

**Figure 1:**
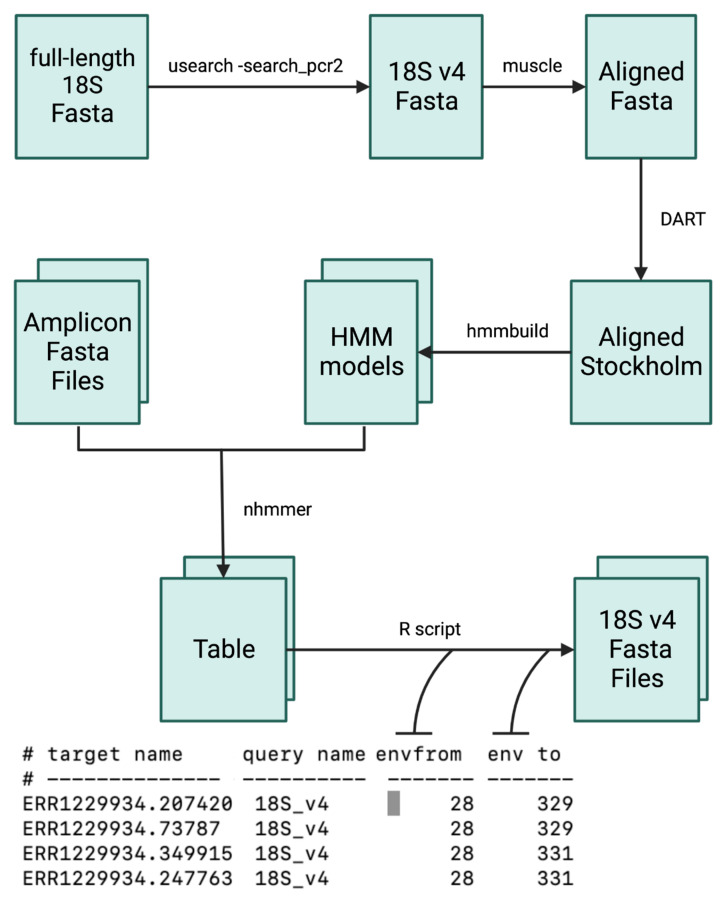
Information scale correction workflow.

We used a multi-step process (Figure 1) to extract and process the 18S rRNA gene v4 region from the full-length 18S rRNA gene amplicon of marine sediment samples. Specifically, we used the Same2 primer set to extract the 18S rRNA gene v4 region, and then employed the USEARCH v11.0.667_i86linux64 [30] search_pcr2 function to obtain the sequences as a fasta file. The sequences were then aligned using MUSCLE v5.1.0 [31] and converted into Stockholm format using DART v1.40 (accessed on 26 December 2021 https://github.com/ihh/dart). Next, we used the resulting alignment file to train an HMM using the HMMER v3.3.2 [32] hmmbuild function. We then treated the resampled data using the 18S rRNA gene v4 HMM model with the HMMER v3.3.2 nhmmer function and output the results in table format. To extract the sub-region sequences, we utilized the Biostrings package v2.66.0 [33]. All the models and scripts related to information scale correction treatment were saved as a reproducible document on GitHub (accessed on 27 March 2023 https://github.com/TongZhou2017/VnFinder/blob/master/vignettes/ISC_workflow.Rmd). We also performed the same process using the V-Xtractor software v2.1 (accessed on 26 December 2021 https://github.com/martin-hartmann/V-Xtractor) [14] to compare the detection sensitivity differences. We excluded methods with low sensitivity, as they may bring additional interference and affect the selection bias. Instead, we selected methods with a sensitivity close to 100%, which would not significantly impact the selection bias.

### 2.3. Bioinformatics Analysis

**Figure 2:**
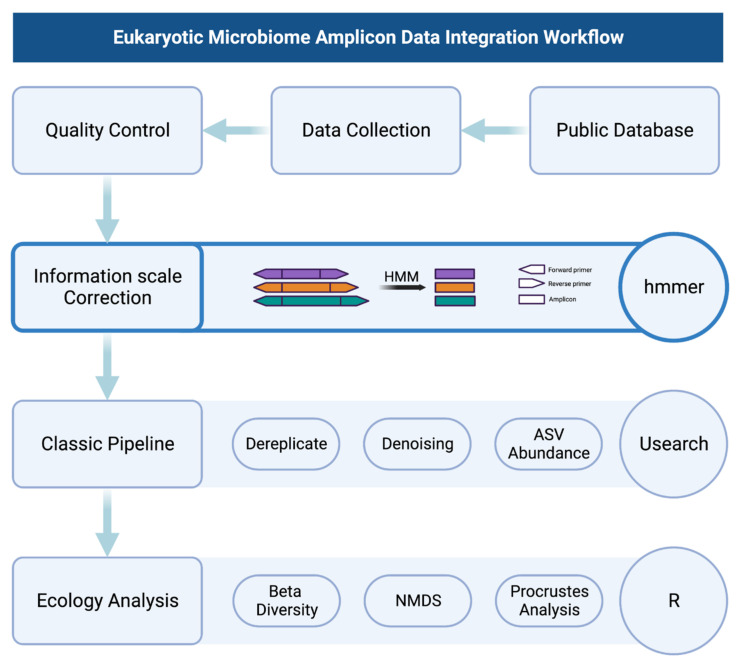
Eukaryotic microbiome amplicon data integration workflow.

In order to evaluate the effectiveness of information scale correction (ISC), we performed an amplicon analysis (Figure 2) on samples before and after ISC using the USEARCH software v11.0.667_i86linux64 (accessed on 1 September 2021 https://drive5.com/usearch/) [30]. This included several steps such as dereplication, ASV denoising, and remapping raw reads to count ASV abundance. The usearch fastx_uniques function is used to remove redundant sequences, without using the relabel parameter, to retain the original sequence IDs for traceability and review of information. Reducing redundancy can reduce computational complexity and improve computational efficiency. The usearch unoise3 function is used to denoise the non-redundant sequences and obtain the ASVs. The denoise methods are more widely accepted than traditional OTU methods [34]. The unoise is commonly used in current denoise methods [35], especially due to its high computational efficiency and suitability for large data computations, compared to dada2 [36]. The otutab function is used to calculate the number of sequences in each sample, representing the abundance information for different ASVs. It should be noted that because the ASV method was used, the zotu parameter was used for the denoised sequence. The resulting ASV abundance table (Table S1) is an important basis for all subsequent calculations. The rows of the table represent ASV IDs, the columns represent samples before and after correction, and the values in the table represent sequence counts.

### 2.4. Statistical Analysis

Based on the ASV abundance table, the microbial community structure differences between each paired group were calculated. The differences were used to assess the effect of ISC. Due to the short sequence length (~300 bp) after processing, the information scale is insufficient to construct a stable phylogenetic relationship, so using the Bray–Curtis index directly is more suitable than the UniFrac distance [6]. The distance between samples was expressed using the NMDS dimensionality reduction method based on Bray–Curtis index and merged using the Procrustes analysis [37]. Procrustes analysis is a statistical method used to compare and merge two sets of multivariate data. It is commonly used in ecological and biological studies to compare community structures between different samples. The M2 value in Procrustes analysis represents the goodness-of-fit between the two sets of data being compared. It measures the degree of similarity between the two sets of data by quantifying the difference between the original configuration of one set of data and its rotated, translated, and scaled version that best matches the other set of data. A value of 0 for M2 indicates a perfect fit between the two sets of data, whereas a larger value indicates a poorer fit. The larger the M2 value, the greater the dissimilarity between the two sets of data being compared. In ecological studies, a significant difference in M2 values between two sets of data indicates that the community structures in the two samples are significantly different. Conversely, a non-significant difference in M2 values indicates that the community structures are similar. 

To evaluate the effectiveness of the correction on each sample in the dataset, we calculated the change in clustering effect before and after treatment. This was done by measuring the distance between each point and the medoids of all samples before treatment. The sample medoids (*m*) was calculated by the pam function in the cluster package v2.1.4 using the pre-treatment data. The effectiveness of the correction (ΔE) was expressed using the difference between the pre- (*b*) and post-treatment (*a*) distances (Equation (1)). The Euclidean distance was calculated by the pointDistance function in the raster package v3.6-20.

(1) $$\Delta E=d\left(m,a\right)-d\left(m,b\right)$$

To evaluate the effectiveness of the correction at the overall level in a dataset, it is necessary to consider the variation in the effectiveness of correction among different samples. Some samples may show significant improvement in microbial community structure similarity after correction, while others may not. To represent the effectiveness of the correction at the overall level in a dataset, a statistical test is performed to assess whether the correction effect is significant, and the average difference in the effectiveness of the correction between paired datasets is calculated. A paired *t*-test was used to test whether a vector of distance difference of the two samples differed from 0. If the distance is shortened (negative), it indicates that the similarity between the samples is improved after correction. Otherwise, it indicates that the correction is invalid. The average effectiveness ($\bar{E}$) of the distance difference between the two groups of samples indicates the effectiveness of the correction at the overall level (Equation (2)). Although the M2 value from Procrustes analysis indicates changes at an overall level, it is not directional for the community structure to become closer or more dispersed.

(2) $$\bar{E}\left(g_{1},g_{2}\right)=\bar{\Delta E_{1}}-\bar{\Delta E_{2}}$$

The effectiveness of the correction is a relative value between each paired sample, corresponding to the difference between the amplified regions of each paired sample. The correlation between the difference in community structure and the difference in the amplified region was counted. It is used to explore whether the difference in community structure is higher as the amplification region becomes longer. If true, it indicates a better correction for longer amplicons and also indicates a stronger incomparability of the original sample. This incomparability is caused by information scale rather than by primer specificity.

## 3. Result

### 3.1. Correction Effect Comparison

**Figure 3:**
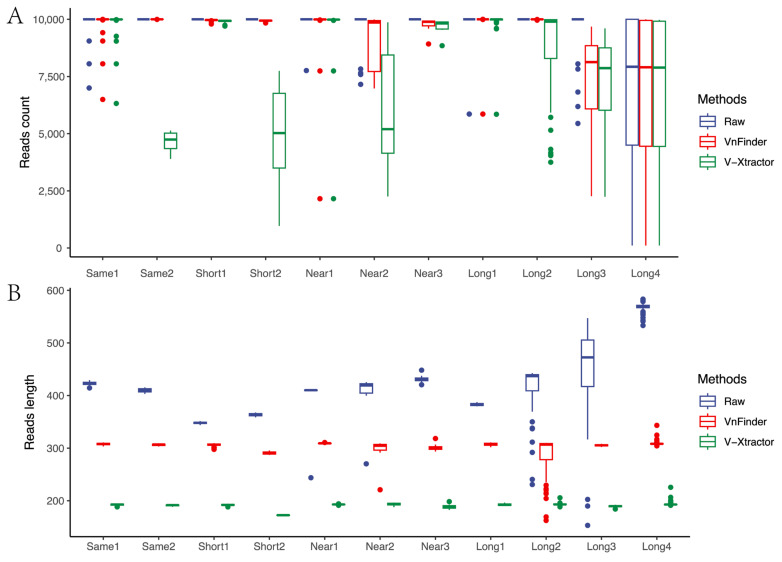
Information scale correction efficiency. (**A**) corrected reads number. (**B**) corrected read length.

Our method outperformed V-Xtractor with a sensitivity close to 100% across all datasets, especially in Same2, Short2, and Long2 datasets, where V-Xtractor exhibited unstable fluctuations in sample extraction efficiency (Figure 3A). V-Xtractor detects the target region based solely on the primer region, leading to an unstable performance in datasets with a loss of primer region information. Furthermore, V-Xtractor uses an extremely short target 18S rRNA gene v4 region (~200 bp), leading to a significant loss of sequence information (Figure 3B), which can result in over-correction. In contrast, our method effectively corrected the scale range of 340~580 bp into about 300 bp, which is stable and preserves most information even for the shortest dataset (Short1). Although Long type samples exhibit the most significant changes in data scale between the blue and red boxes, our method exhibited significantly stable performance based on the complete high-variance region feature, even though the raw region length was not equal to the theoretical amplicon length (Table 1). We also tested the performance of two methods in public database, such as Silva and PR2 (Table 2). These are manually checked reference database. For Silva, our method detected v4 region in 98.32% of the total reads. For PR2, we only detected 88.38%, which might have been because of the low full-length data included in this database. In both these public databases, our method detected more than V-Xtractor. Therefore, using our method for information scale correction avoids primer bias problems and ensures minimal loss of information while avoiding over-correction.

We evaluated the performance of our method in comparison to V-Xtractor in detecting the target region across various datasets. Our results demonstrate that our method outperformed V-Xtractor in terms of sensitivity, with a sensitivity close to 100% across all datasets, particularly in Same2 (on average 9999.304 reads extracted), Short2 (9934.75 reads), and Long2 (9997.795 reads) datasets, where V-Xtractor exhibited unstable fluctuations (4651.696 reads in Same2, 4906 reads in Short2, and 8627.864 reads in Long2) in sample extraction efficiency (Figure 3A). One of the main reasons for the unstable performance of the V-Xtractor is its reliance solely on the primer region for detecting the target region. This approach can lead to an unstable performance in datasets with a loss of primer region information. In addition, V-Xtractor uses a short target 18S rRNA gene v4 region (~200 bp), resulting in a significant loss of sequence information (Figure 3B) that can lead to over-correction. In contrast, our method corrected the scale range of 340~580 bp into about 300 bp effectively, resulting in stable and preserved information, even for the shortest dataset (Short1). Although the Long type samples exhibit the most significant changes in data scale between the blue and red boxes (Figure 3B), our method exhibited stable performance based on the complete high-variance region feature, even though the raw region length was not equal to the theoretical amplicon length (Table 1).

Therefore, our method avoids primer bias problems and ensures minimal loss of information while avoiding over-correction. Our approach effectively corrects the information scale of the datasets, preserving most of the information and providing stable performance, making it a promising alternative to V-Xtractor for target region detection. Our findings suggest that our method can be a valuable tool for detecting the target region in various datasets with high accuracy and minimal loss of information.

### 3.2. Corrected and Non-Differential Effects

**Figure 4:**
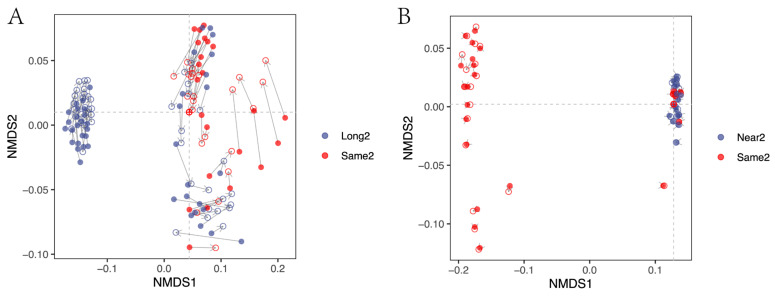
(**A**) Paired comparison of corrected (Long2 vs. Same2) and (**B**) non-differential (Near2 vs. Same2) effects using Procrustes analysis in the Non-metric Multi-dimensional Scaling (NMDS) space. The filled point is for pre-treatment samples; the hollow circle is for post-treatment samples. The red point in the crossing of two dashed lines is the medoids. The M2 value is calculated by Procrustes analysis to evaluate the overall difference between the two datasets. The *p*-value is calculated by *t*-test with H0: there is no difference in the vector of the effectiveness of the correction (ΔE) compared to 0.

Direct comparison of the Bray–Curtis distance has a limitation in that it only considers the pairwise relationships between samples. Some samples have a relatively large change in the two-two relationship (Figure 4A), but the change is small relative to the medoids. With NMDS, the position relationship of the whole sample can be calculated. Therefore, the NMDS method can better reflect the phenomenon of aggregation or distancing between samples. With the Same type samples as the baseline, Long type treatment occurred with inter-sample similarity enhancement. The results of the Procrustes analysis (Figure 4A) indicate the existence of an overall relative relationship changed before and after correction (M2 = 0.305, *p* < 0.001). Notably, some points in the lower right quadrant exhibited a large position change but had an insignificant distance change relative to the original sample medoids. Relative to the original sample medoids, a definite convergence effect occurred for 80.6% of sample points in the corrected sample. Overall, the community structure similarity between the two groups of samples was improved after correction, indicating the effectiveness of ISC treatment for Long and Same type samples. The comparison of data with a difference in amplicon length of over 50 bp between the Long and Same types demonstrated that ISC treatment can correct community structure differences caused by information scale differences, thereby increasing the comparability of samples.

In contrast, the comparison of another dataset showed a different result. The overall relative position change of Near type (Figure 4B) was not significant, and the change of samples was insignificant (M2 = 0.000418, *p* = 0.902). The community structure similarity before and after ISC treatment did not change for the Near and Same types, indicating the ineffectiveness of ISC treatment for these samples. The comparison of data with a difference in amplicon length of less than 50 bp between the Near and Same types demonstrated that there was no difference in community structure after ISC treatment for data with minor information scale differences. This indicates that ISC treatment does not have an over-correction problem, and for the datasets without technical factor caused batch effects, ISC treatment will not change the microbial community structure.

As expected, the amplicon with a larger information scale produced a more remarkable change in the similarity of the community structure after correction. This change tends to be similarity-enhancing, indicating that including information scale correction as a part of big data integration analysis is necessary. 

### 3.3. Correction Effect in Relation to Amplicon Region

**Figure 5:**
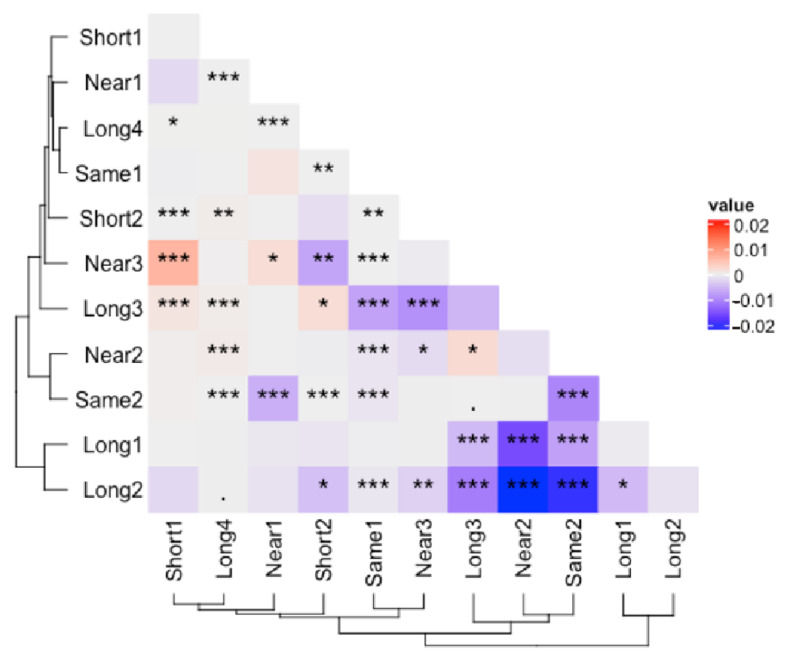
The average effectiveness of the correction in overall level ($\bar{E}$) for community structure similarity improvement. The color scheme distinguishes between increased (blue) and decreased (red) similarity resulting from the correction. Statistical significance is represented by asterisks, with *** indicating a *p*-value < 0.001, ** indicating a *p*-value < 0.01, * indicating a *p*-value < 0.05, and ‘.’ indicating a *p*-value < 0.1.

A two-by-two comparison of the samples of all types revealed that the most significant correction existed mainly between Long-Same and Long-Near (Figure 5). At the same time, there was no significant difference between Same and Near. There was a considerable variation in the Short type compared to other types, such as Short1-Short2, Short1-Near3, Short1-Long3, and Short2-Long4. This indicates that the minimum length amplicon should be chosen as the baseline in the information scale correction. Otherwise, the amplicon with too small information scale should be filtered out to maintain stability. A negative correlation between the information scale and the effectiveness of the correction can still be found in the 11 datasets. This indicates that the effect of information scale correction is real. Additionally, there is a tendency for the batch effect to increase with the increase in the information scale. It can be inferred that as the information scale increases, such as the current long-read amplicon, the batch effect caused by the information scale will mask the community structure similarity existing in the sample itself, which in turn causes the non-comparability between amplicon data of different lengths.

## 4. Discussion

The most significant contribution of this study is the proposal of a novel Information Scale Correction (ISC) method. This method has the advantage of higher sensitivity than the existing 18S rRNA gene v4 region extraction methods (Figure 3A), which ensures that the ISC process does not cause primer bias interference, unlike the in-silico PCR method based on primers. We effectively performed ISC on amplicons with varying lengths (Figure 3B).

The amplicon with a large difference in information scale tends to significantly improve sample similarity (Figure 5). For example, Long1 and Long2 are more than 50 bp longer than Same2 and Near2, and their similarity improved after ISC. Figure 4A shows more details, where most samples were closer to the medoids after ISC. Amplicons with similar lengths and primer set positions typically have no information scale difference, so ISC would not bring additional disruptions to such amplicons (Figure 4B). Information scale correction is a subtype of batch effect correction. The critical characteristic of batch effect correction is to reduce the non-related differences caused by technical factors while preserving the original difference information between samples [6,15]. Most correction results between different scale types are significantly changed (Figure 5). Furthermore, it is essential to note that for samples with similar information scales (Figure 4B), the correction effect of ISC is not significant. This suggests that correction is not causing unnecessary interference, i.e., over-correction. It is usual for data correction to result in changes, but it is crucial to correct only where changes are expected (Figure 4A) and not over-correct where changes should not occur (Figure 4B). This is the hallmark of an effective correction method. This indicates that when integrating datasets of varying read lengths, it is necessary to incorporate the data processing step of information scale correction to reduce the batch effect caused by information scale differences.

Our study demonstrates that long-read amplicon sequencing presents challenges in integrating large datasets, not only due to amplification specificity but also due to differences in information scale. We have observed in our lab analysis and in other public datasets [4], that long-read amplicon sequencing using PacBio or Nanopore platforms results in ASV abundance tables with many low-abundance ASVs, making it difficult to directly integrate analysis with next-generation sequencing datasets. In our data, we also observed a similar phenomenon. Long4 is the longest amplicon data obtained using Roche 454 sequencing method among the 11 datasets (Table 1). After applying ISC, there was a decrease in community similarity (Figure 5), such as in the cases of Long4-Near2 and Long4-Long3. This could be because when there is a large difference in sequence lengths, evaluation methods based on Bray–Curtis dissimilarity are not suitable. This is because a large degree of ISC can result in significant changes in the ASV denoising results, which can be observed in ASVs that are closely related based on phylogenetic evaluation methods, such as UniFrac distance [6]. However, due to the lack of full-length sequences for eukaryotic microorganisms, unlike the rich accumulation of full-length sequences in prokaryotic organisms, it is difficult to integrate amplicon data with different information scales into a unified phylogenetic tree based on full-genome or 18S full-length sequences.

Meanwhile, another similar limitation of our study is the lack of a well-established metabarcoding molecular ecology database, such as the PR2-primer database [18], to find primer information. Although the dataset was relatively small, it had a wide range of amplicon lengths, and each category of amplicon had at least two datasets. Our results showed that information scale correction was effective in these datasets, particularly for the long amplicon, as shown in Figure 5. We hope that in the future, a metabarcoding database containing information on primers, raw data, and environmental information will be constructed, enabling the validation of larger datasets. Such a database would be especially useful for large-scale microeukaryote ecology, where a reference universe [6], consisting of a large amount of historical data produced by various sequencing platforms, including Sanger sequencing, 454 sequencing [38], IoT sequencing [39], Illumina HiSeq [40], and MiSeq [19], as well as the increasing number of PacBio and Nanopore sequencing results, is essential.

## 5. Conclusions

This study addresses the challenges posed by differences in information scale between amplicon datasets. We propose a novel information scale correction (ISC) method for integrating varying-length amplicon datasets. We trained a Hidden Markov model (HMM) by using the complete 18S rRNA gene v4 region data. We evaluated the performance of the ISC method in the Silva and PR2 databases. Our method achieved an identification rate of 98.32% in Silva and 88.38% in PR2, respectively. In contrast, V-Xtractor, the most popular tool for sub-region extraction, has only an identification rate of 95.83% in Silva and 76.62% in PR2. We tested with 11 amplicon datasets which were divided into four types by length and overlap relationship: Same, Short, Near, and Long. The results showed that after ISC processing, the community structure similarity of the amplicons with significant length differences (Long vs. Same) improved, indicating that the ISC method effectively corrected the information scale difference. Moreover, the community structure of the amplicons with similar lengths (Near vs. Same) did not change significantly, indicating that the ISC method did not introduce strong biases. Nonetheless, for the shortest amplicons (Short), significant fluctuations were observed in the community structure after correction, suggesting that the impact of the shortest data should be considered during information scale correction. The ISC method provides a feasible approach for integrating large amplicon datasets of eukaryotic microorganisms, which is helpful for advancing our knowledge of microbial ecology.

## Figures and Tables

**Table 1:** Data and primers information.

Group ID	Forward Primer	Start Position	Reverse Primer	End Position	Sample Size	Source (ID)	Reference
Same1	CCAGCASCYGCGGTAATTCC	564	ACTTTCGTTCTTGAT	980	120	EBI-ENA (PRJNA508517)	[19]
Same2	CCAGCASCYGCGGTAAT	564	ACTTTCGTTCTTGATYRA	980	23	NCBI-SRA (PRJEB24415)	[20]
Short1	CYGCGGTAATTCCAGCTC	571	TCYDAGAATTYCACCTCT	914	73	NCBI-SRA (PRJEB21047)	[21]
Short2	TTAAAAAGCTCGTAGTTG	616	AAGAAGACATCCTTGGTG	963	27	NCBI-SRA (SRR5189947)	[22]
Near1	GCGGTAATTCCAGCTCCAA	573	ACTTTCGTTCTTGATYRR	980	33	EBI-ENA (PRJEB26288)	[23]
Near2	CCAGCASCCGCGGTAATWCC	564	AKCCCCYAACTTTCGTTCTTGAT	988	17	NCBI-SRA (PRJNA368621)	[24]
Near3	CCAGCASCCGCGGTAATWCC	564	TCTGRTYGTCTTTGATCCCYTA	1002	12	EBI-ENA (PRJEB12534)	[25]
Long1	CGGTAAYTCCAGCTCYAV	574	CCGTCAATTHCTTYAART	1149	62	NCBI-SRA (SRP071862)	[26]
Long2	GTGCCAGCAGCCGCG	561	TTTAAGTTTCAGCCTTGCG	1138	44	NCBI-SRA (PRJNA482746)	[27]
Long3	GGCAAGTCTGGTGCCAG	551	TCCGTCAATTYCTTTAAGT	1149	36	EBI-ENA (PRJEB24755)	[28]
Long4	CGGTAATTCCAGCTCCAATAGC	574	CACCAACTAAGAACGGCCATGC	1293	150	NCBI-SRA (SRP101780)	[29]

**Table 2:** Extraction performance in public databases.

	Silva	PR2
Version	18S v123	v4.14.0
Total reads	138,553	197,602
Full length (>1600 bp)	112,110 (80.91%)	94,283 (47.71%)
Partical (<1000 bp)	293 (0.21%)	48,002 (24.29%)
v4 by VnFinder	136,228 (98.32%)	174,646 (88.38%)
v4 by V-Xtractor	132,770 (95.83%)	151,393 (76.62%)

## Data Availability

The ASV abundance table data are presented in the Electronic Supplementary Material (https://www.mdpi.com/article/10.3390/microorganisms11040949/s1). Other raw data from this study will be provided by the authors upon request, without restrictions.

## Supplementary Materials

The following supporting information can be downloaded at: https://www.mdpi.com/article/10.3390/microorganisms11040949/s1, Table S1: ASV abundance.
